# Regeneration of periodontal intrabony defects using platelet-rich fibrin (PRF): a systematic review and network meta-analysis

**DOI:** 10.1007/s10266-024-00949-7

**Published:** 2024-05-21

**Authors:** Fábio França Vieira e Silva, Luis Chauca-Bajaña, Vito Carlo Alberto Caponio, Kareelend Andreina Segura Cueva, Byron Velasquez-Ron, Maria Elena Padín-Iruegas, Lays Lamolha Almeida, Alejandro Ismael Lorenzo-Pouso, José Manuel Suárez-Peñaranda, Mario Pérez-Sayáns

**Affiliations:** 1 Faculty of Medicine and Dentistry, Oral Surgery and Implantology Unit (MedOralRes, Oral Medicine, Universidade de Santiago de Compostela. Health Research Institute of Santiago de Compostela (IDIS), Santiago de Compostela University Hospital Complex, Rúa da Choupana, S/N, 15706 Santiago de Compostela, A Coruña Spain; 2 Periodontics and Implantology Oral Research. College Dentistry, Ecuador. Faculty of Medicine and Dentistry, University of Guayaquil, Oral Medicine, Oral Surgery and Implantology Unit, Universidade de Santiago de Compostela, Santiago de Compostela, A Coruña Spain; 3 Department of Clinical and Experimental Medicine, University of Foggia, 71100 Foggia, Italy; 4 Oral Surgery, Department of Dentistry, University of Guayaquil, Quito-Ecuador, Ecuador; 5 Dental Prosthesis Department Research. College Dentistry, University of the Americas. UDLA. Av, Colon y 6 de Diciembre, Campus Colón, Quito-Ecuador, Ecuador; 6 Human Anatomy and Embryology Area, Department of Functional Biology and Health Sciences, Faculty of Physiotherapy, University of Vigo, 36001 Pontevedra, Spain; 7 Department of Dental Medicine, Federal Fluminense University, Rio de Janeiro, 28625650 Brazil; 8 Oral Medicine, Oral Surgery and Implantology Unit (MedOralRes), Faculty of Medicine and Dentistry, University of Santiago de Compostela, 15782 Santiago de Compostela, Spain; 9 Faculty of Medicine and Dentistry, Oral Surgery and Implantology Unit (MedOralRes, Oral Medicine, Universidade de Santiago de Compostela. Health Research Institute of Santiago de Compostela (IDIS), Instituto de los Materiales de Santiago de Compostela (iMATUS), Avenida Do Mestre Mateo, 25, 15782 Santiago de Compostela, A Coruña Spain

**Keywords:** Furcation defects, Guided periodontal tissue regeneration, Platelet-rich fibrin, Periodontal disease, Randomized clinical trial

## Abstract

One of the most promising approaches to correct periodontal bone defects and achieve periodontal regeneration is platelet-rich fibrin (PRF). This systematic review and meta-analysis aimed to evaluate the regeneration of periodontal bone defects using PRF compared to other regenerative treatments. The data search and retrieval process followed the PRISMA guidelines. An electronic search of MEDLINE, Cochrane, and PubMed databases was performed, selecting exclusively randomized clinical trials where the following were measured: probing depth reduction (PD), clinical attachment level gain (CAL), and radiographic bone fill (RBF). Out of 284 selected articles, 32 were chosen based on inclusion criteria. The use of platelet-rich fibrin (PRF) + open flap debridement (OFD), PRF + metformin, PRF + platelet-rich plasma (PRP), and PRF + OFD/bone graft (BG) significantly reduced PD and improved CAL and RBF. However, the combination of PRF + BG, PRF + metformin, and PRF + STATINS reduced CAL. The intervention of PRF combined with different treatments such as metformin, OFD, PRP, BG, and STATINS has a significant impact on improving PD and CAL. The use of PRF significantly improved the regeneration of periodontal bone defects compared to other treatments.

**Supplementary Information:**

The online version contains supplementary material available at 10.1007/s10266-024-00949-7.

## Introduction

Periodontal disease is a multifactorial chronic disease that destroys the supporting tissues of the tooth (bone, cement, and periodontal ligament) [1, 2]. Studies indicate that 47% to 50% of the adult population has periodontal disease, and 38.5% are in moderate to severe stages of the disease (stage III or stage IV) [3–5]. The main objective of periodontal disease treatment is to suppress inflammation by controlling infection, although it is also possible to achieve partial periodontal regeneration in certain cases [6]. Periodontal regeneration is the reconstruction of the part of the tissues that suffered damage and may be accompanied by the loss of supporting tissues [7]. The pathological progression of periodontal disease will generate bone resorption causing vertical and/or horizontal bone defects [8]. Periodontal regeneration seeks to promote the growth of new tissues and the proper formation of periodontal structures to restore the health and function of teeth affected by periodontal diseases [9]. Currently, there are surgical techniques and regenerative materials such as guided tissue regeneration, growth factors, enamel matrix derivates, bone grafts, barrier membranes, and mesenchymal stem cells that are used to repair and regenerate periodontal tissue, bone defects, atrophic alveolar ridge, and furcation defects [10]. In regenerative medicine, the correction of periodontal intrabony defects using platelet concentrates (PC) has been studied [11]. Platelet-rich plasma (PRP) releases growth factors for tissue healing and regeneration [12, 13], demonstrating that anticoagulants interfere with the angiogenic and regenerative responses measured by the plates [14]. In regenerative medicine and dentistry, a second-generation platelet concentrate called platelet-rich fibrin (PRF) has been introduced, which does not require anticoagulants [15, 16]. Periodontal intraosseous defects are considered to have good regeneration potential [17]. Several randomized clinical trials (RCTs) have been reported using PRF since one of the advantages is the formation of dense fibrin clots with platelets and leukocytes, which favors a more prolonged release over time [18, 19]. In addition, the use of PRF in periodontal bone defects has been studied in several systematic reviews, which conclude that it favors the healing of periodontal tissues [20]. The present systematic review and meta-analysis aim to evaluate the regeneration of periodontal bone defects using PRF in comparison with other regenerative treatments.

## Material and methods

Prospero database was originally accessed in May of 2023 and the protocol for this systematic review and meta-analysis was submitted and successfully registered: CRD42023431418. Preferred Reporting Items for Systematic Reviews and Meta-analyses (PRISMA) guidelines were followed [21].

## PICO question

*"Is the regeneration of periodontal intrabony defects with PRF more effective than other techniques?"* (P: Articles with studies of periodontal intrabony defects in humans were evaluated; I: Intervention, regeneration of periodontal intrabony defects performed with PRF, alone or in combination with other biomaterials; C: Comparison of the different regeneration results of the supporting periodontal tissue with different regenerative materials; O: Observation, the amount of periodontal regeneration was compared, measured by probing depth, clinical attachment level, and alveolar bone in the periodontal defect). 

## Search strategy and database screening

The Rayyan QCRI program (Qatar Computing Research Institute, Doha, Qatar) was used to identify eligible articles. The search strategy included the screening of different databases, such as MEDLINE through PubMed, EMBASE through OVID, Web of Science, Scopus, Cochrane Library, Clinical Trials, the five WHO regional bibliographic databases (AIM, LILACS, IMEMR, IMSEAR, WPRIM), and Conference Proceedings Citation Index. A combination of keywords and terms was set and adjusted for each database. The simple model of keywords used were: "Platelet-rich fibrin", "Platelet-rich plasma", "Periodontal attachment loss", "Alveolar bone loss", "Guided tissue regeneration", "PRF", "Periodontal bone defects", "Furcation defects”; AND/OR were also included when searching. This process was complemented by a manual search (peer-reviewed journals with related content).

## Eligibility criteria

### Inclusion criteria

1. Studies on the regeneration of bone defects with PRF in humans; 2. Studies of randomized clinical trials with PRF alone or with other regenerative biomaterials used locally to correct periodontal defects; 3. Regeneration data probing depth, clinical attachment level, and alveolar bone; 4. Studies published in English.

## Exclusion criteria

1. Studies including patients with diabetes; 2. Studies including patients with osteoporosis; 3. Studies with patients with genetic modifications; 4. Preclinical in vitro or animal studies; 5. Periodontal regeneration without using PRF; 6. Studies that are not randomized clinical trials; 7. Clinical cases, cohort, and retrospective studies; 8. Studies for which measurements and standard deviation data were missing; 9. Reviews, systematic reviews, and meta-analysis.

## Studies screening and data extraction

An ad-hoc extraction sheet was created and filled independently by three investigators (LC, KS, and MPS) using a customized data sheet. Any doubts that existed between the three investigators were resolved by three investigators (BVR, CCC, and REV) who were unaware of the study hypothesis. The following data were recorded: First author, year, study design, type of study, number of people, gender, mean age, types of defects, intervention groups, control group, smokers, conclusions, mean difference (MD) in probing depth (PD), clinical attachment level (CAL), alveolar bone, spin system, the volume of blood drawn, and spin parameters (Table 1; Supplementary Table 1).

**Table 1:** General overview of the included studies

Author	Study design	Follow-up	Sample size	Gender	Average age	Types of defects	Intervention group	Control group	Smokers	Conclusion
*OFD vs PRF*										
Sharma and Pradeep 2011 [15]	RCT parallel	9 months	42	24 M 18 F	35.3	3 walls	T: 28, OFD + PRF	C: 28 OFP	No	Greater reduction in probing depth, CAL gain, and bone fill at PRF-treated sites with conventional open-flap debridement
Thorat et al. 2011 [16]	RCT parallel	9 months	32	20 M 12 F	30.7	2 and 3 walls	T: 16, OFD + PRF	C: 16, OFD	No	Greater reduction in PD, more CAL gain, and greater filling of intrabony defects in the PRF-treated sites
Rosamma et al. 2014 [17]	TC split mouth	12 months	15	6 M 9 F	29.5	2 and 3 walls	T: 15, OFD + PRF	C: 15, OFD	No	Clinically, the use of PRF in both gel and membrane forms is more effective than open flap debridement alone in the management of horizontal periodontal defects
Ajwani et al. 2015 [18]	RCT	9 months	20	10 M 20 F	30.5	2 and 3 walls	T: 20, OFD + PRF	C: 20, OFD	No	The complementary use of PRF with OFD significantly improves defect filling compared to OFD alone
Bajaj et al. 2017 [19]	RCT	9 months	17	9 M 8 F	29.7	2 and 3 walls	T: 27, OFD + PRF	C: 27, OFD	No	There is greater bone fill in the PRF-treated areas with conventional OFD than with conventional OFD alone
Patel et al. 2017 [20]	RCT	12 months	13	4 M 9 F	44	2 and 3 walls	T: 13, OFD + PRF	C: 13, OFD	No	The use of PRF with conventional OFD can potentially be used in the treatment of periodontal bone defects
Pradeep et al. 2017 [21]	RCT	9 months	62	34 M 28 F	39.7	3 walls	T1: 19, OFD + PRF, T2: 20, OFD + PRF + HA	C: 18, OFD	No	PRF results in significant improvements of clinical parameters. When added to PRF, HA augments the regenerative effects seen with PRF in 3-wall IBD treatment
Thorat et al. 2017 [22]	RCT	12 months	15	7 M 8 F	25	3 walls	T: 15, OFD + PRF	C: 15, OFD	-	The use of PRF significantly improves the clinical and radiographic results of open flap debridement in the treatment of periodontal intrabony defects in patients affected by localized aggressive periodontitis
*BG vs PRF*										
Mathur et al. 2015 [23]	RCT	6 months	25	14 M 11 F	39.7	2 and 3 walls	T: 19, OFD + PRF	C: 19, OFD + ABG	No	The use of PRF or ABG was effective in the treatment of three-wall periodontal intrabony defects with uneventful healing of the sites
Shah et al. 2015 [24]	RCT	6 months	20	-	-	2 and 3 walls	T: 20, OFD + PRF	C: 20, OFD + DFDBA	No	PRF has shown significant results after 6 months, which is comparable to DFDBA for periodontal regeneration
Chadwick et al. 2016 [25]	RCT	6 months	36	20 M 16 F	54.9	2 and 3 walls	T: 17, OFD + PRF	C: 19, OFD + DFDBA	Yes	Treatment of periodontal intrabony defects with DFDBA or PRF resulted in a significant gain in CAL and bone fill after 6 months of healing, with no significant differences between materials
Galav et al. 2016 [26]	RCT	9 months	20	-	45	2 and 3 walls	T: 20, OFD + PRF	C: 20, OFD + ABG	No	Both ABG and PRF can be used in predictable ways to rebuild lost periodontal structures as indicated by PPD reduction and RAL gain. However, in terms of filling bone defects, ABG produces a more definitive result than PRF
Yajamanya et al. 2017 [7]	RCT	9 months	32	-	-	2 and 3 walls	T1: 28, OFD + BioG T2: 28, OFD + PRF	C: 28, OFD	No	Significant improvements in clinical parameters and radiographic results with PerioGlas ® and autologous PRF to treat periodontal intrabony defects compared to OFD alone
*BG vs BG* + *PRF*										
Bansal and Bharti 2013 [27]	RCT	6 months	10	-	-	-	T: 10, OFD + DFDBA + PRF	C: 10, OFD + DFDBA	-	Combining PRF with DFDBA demonstrated better results in probing depth reduction and clinical attachment level gain compared to DFDBA alone in the treatment of periodontal intrabony defects
Elgendy and Abo Shady 2015 [28]	RCT	6 months	20	-	44	-	T: 20, OFD + HA + PRF	C: 20, OFD + NcHA	Yes	NcHA bone grafting in combination with PRF demonstrated clinical advantages beyond those achieved by NcHA alone
Agarwal et al. 2016 [29]	RCT	12 months	30	15 M 15 F	52	2 and 3 walls	T: 30, OFD + DFDBA/PRF	C: 30, OFD + DFDBA	No	Combination of PRF and DFDBA is more effective than DFDBA with saline for the treatment of infrabony periodontal defects
Naqvi et al. 2017 [30]	RCT	9 months	10	7 M 3 F	N/R	2 and 3 walls	T: 10, OFD + BioG + PRF	C: 10, OFD + BioG	No	The results of this study showed that BioG putty alone from both groups and the combination of PRF and BioG putty are effective in the treatment of subbony periodontal defects
Sezgin et al. 2017 [31]	RCT	6 months	15	8 M 7 F	N/R	2 and 3 walls	T: 15, OFD + ABBM + PRF	C: 15, OFD + ABBM	No	Both therapies are effective in the treatment of intrabony defects
Liu et al. 2021 [32]	RCT	12 months	15	4 M 11 F	36.0 ± 8.6	-	T: 14 BPBM + PRF	C: 14 BPBM	No	The BPBM-PRF complex is clinically more effective and produces better clinical results
Paolantonio et al. 2020 [33]	RCT	12 months	44	15 M 29 F	53 ± 12	1 and 2 walls	T: 22 L-PRF and ABG	C: 22 EMD + ABG	No	The results suggest that the L-PRF + ABG combination treatment of uncontained IBD produces non-inferior results in terms of CAL increase, PPD reduction, GR increase, and DBL increase compared to the EMD + ABG combination
Bodhare et al. 2019 [34]	RCT	6 months	20	11 M 9 F	35.9	2 and 3 walls	T: 20, OFD + Bioactive Glass + PRF	C: 20, OFD + Bioactive Glass	No	The use of Bioactive Glass, when used in combination with PRF, is more effective in gaining CAL, reducing PPD, and achieving greater bone fill compared to BG treatment alone in periodontal and intrabony defects. it is indicative of improved periodontal regeneration
*BM vs PRF*										
Pham 2021 [35]	RCT	12 months	30	22 M 8 F	47.9	2 and 3 walls	T: 30 PRF + OFD	C: 30 GRUPO 2 BM + GTR Y 30 GRUPO 3 OFD	No	Compared to GTR, PRF yielded comparable periodontal tissue healing and treatment results in terms of improvements in clinical and radiographic parameters. Compared with OFD alone, PRF also significantly improved these parameters in the treatment of intrabony defects
Ustaoğlu et al. 2020 [36]	RCT	9 months	45	23 M 22 F	40 ± 8.37	Intrabony defects associated with a primary periodontal lesion with secondary endodontic involvement or true combined endodontic-periodontal lesions in single-rooted teeth	T: 15 PRF + OFD	C: 15 BM + GTR GRUPO 2 Y 15 OFD GRUPO 3	No	PRF can give similar successful results to GTR in the treatment of IBD with endo-perio lesions
Panda et al. 2016 [37]	RCT	9 months	18	10 M 8 F	38.1	3 walls	T: 18, OFD + BM + PRF	C: 18, OFD + BM	No	The adjunctive use of PRF in combination with the barrier membrane is more effective in the treatment of intrabony defects in chronic periodontitis compared to the barrier membrane alone
*PRP vs PRF*										
Pradeep et al. 2012 [38]	RCT	9 months	54	27 M 27 F	36.8	3 walls	T1: 17, OFD + PRP T2: 16, OFD + PRF	C: 17, OFD	No	Similar reduction of PD, CAL gain and BF in PRF or PRP treated sites with conventional OFD
*EMD vs PRF*										
Gupta et al. 2014 [39]	RCT	6 months	30	15 M 15 F	-	3 walls	T: 22, OFD + PRF	C: 22, OFD + EMD	No	Both Emdogain and platelet-rich fibrin were effective in regenerating intrabony defects. Emdogain was significantly superior in terms of defect resolution percentage
CsifóNagy 2021 [40]	RCT	6 months	18	9 M 9 F	55,5 ± 14,5	2 and 3 walls	T: 15, OFD + PRF	C: 15 OFD + EMD	No	New generation PRF appears to be as clinically effective as DME during surgical treatment of intrabony defects
*EMD vs EMD* + *PRF*										
Aydemir Turkal et al. 2016 [41]	RCT	6 months	28	14 M 14 F	38.5	1, 2 and 3 walls	T: 25, OFD + EMD + PRF	C: 24, OFD + EMD	No	Both therapies resulted in significant clinical improvement in IBD treatment. The addition of PRF did not improve clinical and radiographic results
*PRF vs PRF* + *metformin*										
Pradeep et al. 2015 [42]	RCT	9 months	120	60 M 60 F	41	3 walls	T1: 30, OFD + PRF T2: 30, OFD + 1% MF + PRF	C1: 30, OFD C2: 30, OFD + 1% MF	No	The study showed that the PRF + 1% MF group was more effective than MF, PRF or OFD alone in the treatment of infrabony periodontal defects
*PRF vs PRF* + *bisphosphonates*										
Kanoriya et al. 2016 [43]	RCT	9 months	90	43 M 47 F	40.3	3 walls	T1: 30, OFD + PRF T2: 30, OFD + PRF/1% ALN	C: 30, OFD	No	Combined approach therapy of PRF + 1% ALN for the treatment of IBD in CP patients showed better clinical parameter outcomes with greater reduction in IBD depth compared to PRF and access therapy alone
*PRF vs PRF* + *statins*										
Martande et al. 2016 [44]	RCT	9 months	96	48 M 48 F	37.6	3 walls	T1: 30, OFD + PRF T2: 30, OFD + PRF + 1,2% ATV	C: 30, OFD	No	1.2% ATV failed to increase the regenerative potential of PRF in periodontal bone defects alone
Pradeep et al. 2016 [45]	RCT	9 months	90	45 M 45 F	35	2 and 3 walls	T1: 30, OFD + PRF T2: 30, OFD + PRF + 1,2%rosuvastatina	C: 30, OFD	No	OFD with rosuvastatin and PRF produces significantly greater periodontal benefits compared to OFD alone or with PRF

General Information. *OFD* Open Flap Debridement; *PRF* Platelet-Rich Fibrin; *CAL* Clinical Attachment Level; *T* Treatment; *C* Control; *HA* Hydroxyapatite; *ABG* Autogenous Bone Graft; *DFDBA* Allogeneic Demineralized Freeze-Dried Bone, *BioG* Bioactive glass; *ABBM* Anorganic Bovine Bone Mineral; *BPBM* Bovine Porous Bone Mineral; *L-PRF* Leukocytes and Platelet-Rich Fibrin; *EMD* Enamel Matrix Derivative; *M* Men; *F* Female; *GTR* Guided Tissue Regeneration; *BM* Barrier Membrane; *PRP* Platelet-Rich Plasma; *PD* Probing Depth; *BF* Bone Filler; *MF* MetFormin; *ALN* Alendronate; *ATV* Atorvastatin; *BG* Bone Graft; *RCT* Randomized Clinical Trials [7, 12, 30–59].

## Assessment of risk of bias (RoB)

Two authors (MS, VM) independently assessed the included reports, using all checklist items of the respective scales. The Cochrane Risk of Bias Tool for Randomized Controlled Trials was used to assess randomized controlled trials [22].

The RoB was classified as "high", "unclear" and "low". These studies were analyzed in five domains: 1. Sequence generation: It was evaluated by taking into account whether or not there were periodontal intrabony defects; 2. Concealment: Baseline characteristics were checked in the test and control groups; 3. Incomplete data: The inclusion of all data, number of patients, number of periodontal intrabony defects, control group, intervention group, patients without systemic disease, probing depth data, clinical attachment level, and alveolar bone was observed; 4. Selective reporting of results: Study protocols and group results were assessed only with PRF or other regenerative materials and other sources of bias; 5. Selective reporting of the results: The intervention groups with PRF in periodontal intrabony defects were compared with the control group, to observe if there were patients with additional medication, different treatments of intrabony defects, the number of interventions performed, and if any people received the same care or not. 

## Statistical analysis

### Qualitative analysis

A systematic review of all the included articles was carried out, determining the characteristics previously defined in the inclusion criteria (see data extraction). Two groups of studies were established: 1. The first group of periodontal intrabony defects with PRF alone or mixed with other types of regenerative materials; 2. The second group only other regenerative materials or without placing any regenerative materials.

Probing depth gain, clinical attachment level, and periodontal bone regeneration were evaluated in each group.

## Meta-analysis

To perform the meta-analysis, the studies were grouped according to their characteristics and results obtained, extracting the name, sample size, mean, and standard deviation of the treatment and control groups, segmented by PD, CAL, and radiographic bone fill (RBF) criteria. With all the data sorted and classified, the statistical software IBM SPSS version 29 was used, the Meta-analysis section with continuous results, where Cohen’s d was used for the effect size with the random effects model, with a 95% confidence interval, with a restricted maximum likelihood estimator (REML), adding the Forest-plot graphs with effect size, standard error, confidence interval limits, P-value, weighting, including the reference line for overall effect size and null effect size, as well as the Funnel-plot graph showing standard error and effect size. According to the results, p-values < 0.05 are considered statistically significant, according to the I2 the tests with values < 25% were classified as low heterogeneity, while values of 25 and < 50% are classified as moderate, but > 50% are considered high heterogeneity.

## Network meta-analysis

To assess the effects of various commercial and non-commercial biomaterials, including information regarding grafting materials that haven’t been directly compared before, an arm-based network meta-analysis was carried out. Network meta-analysis (NMA) is an expansion of the traditional pairwise meta-analysis and can be used to compare many distinct treatments. Using this method, it is possible to synthesize a lot of data, estimate relative efficacy, and rank interventions based on their effectiveness. Results from the data extraction process were combined afterward (network setup command) to fit STATA software. Similarity, transitivity, and consistency were tested as relevant assumptions for network meta-analysis [23]. By analyzing demographic, intervention, comparison, and outcome, the similarity of the included studies was subjectively evaluated [24]. By statistically examining the consistency of the results of direct and indirect comparisons, transitivity was further evaluated [25]. Predictive interval plots and network geometry plots were consequently made. We used the network geometry plot to visualize the network of the various groups and investigate connections among them. The groups are represented by the nodes, and the direct comparisons between groups are shown by the edges. Surface under the Cumulative Ranking Curves (SUCRA) were utilized to determine the relative ranking of groups using probability. To establish a hierarchy of the interventions, SUCRA, a straightforward transformation of the mean rank, accounts for both the location and the variation of the relative treatment effects. The rank of the treatment improves with increasing SUCRA values. For hypothesis testing, a two-tailed p-value of 0.05 was regarded as significant. Clinical studies were used to extrapolate data on the mean difference, standard deviation, and total sample size for each intervention group. Three different NMA included PD, CAL, and RBF, and the relative mean effects and projections for each comparison are shown in the estimated summary effects forest plot, which includes confidence intervals and predictive intervals. NMA was performed using mvmeta network commands in the STATA software suggested by Chaimani et al. [26–29].

## Results

### Study selection

The results of the search are based on the PRISMA guidelines [21]. 284 articles whose abstracts were reviewed for content relevant to the topic under study were identified, from which 252 studies were excluded. Once the critical analysis was completed, 32 articles from a randomized clinical trial met the inclusion criteria for the regeneration of periodontal intrabony defects with PRF alone or with another regenerative biomaterial [7, 12, 30–59]. These data can be found in Supplementary Fig. 1.

## Quality assessment

Articles were assessed using the Cochrane RoB Tool for RCTs [22]. All studies were found to be at low RoB [7, 12, 30–59]. These data can be seen in Supplementary Table 2.

## Meta-analysis

### PD group

**Fig. 1:**
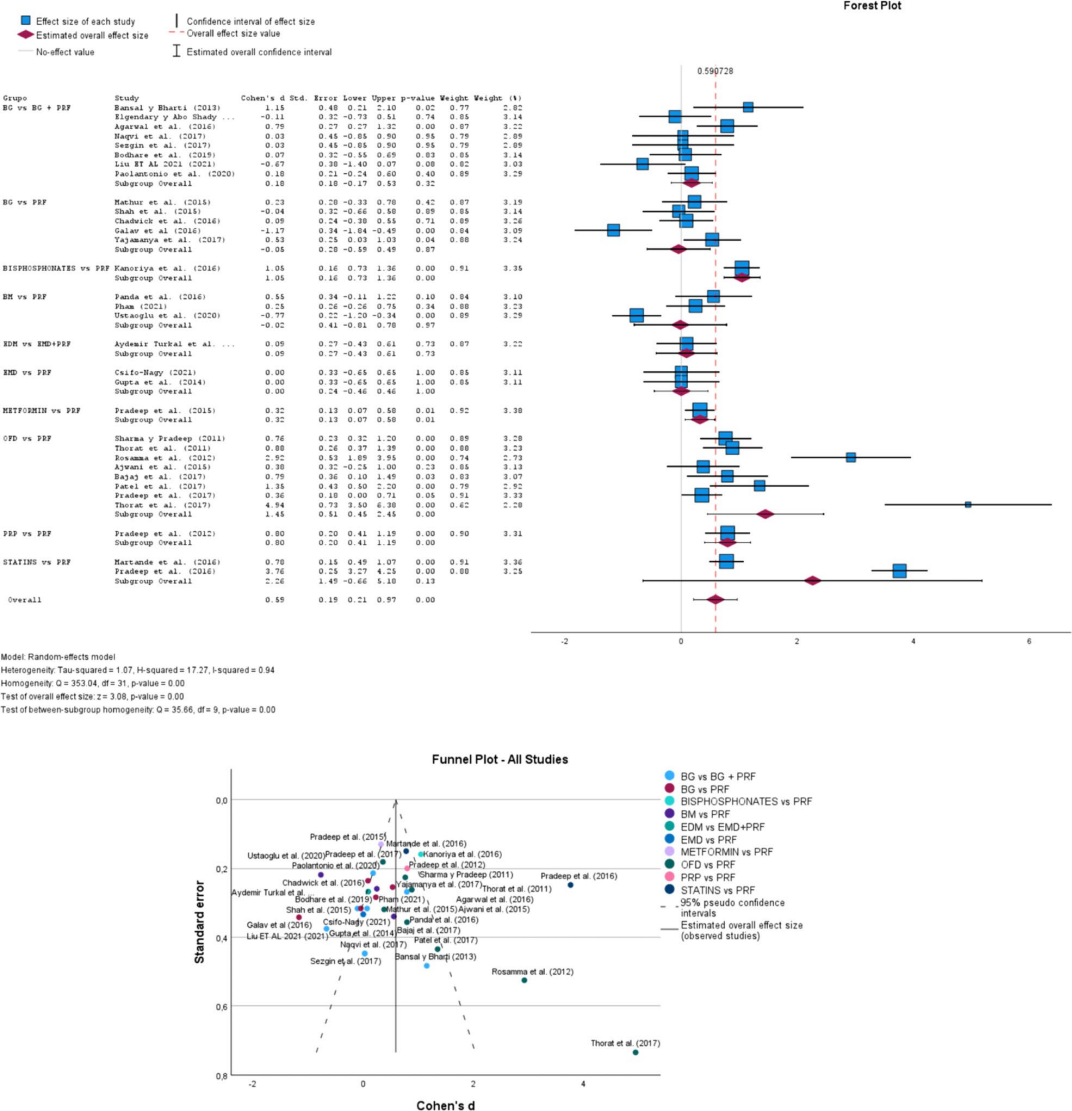
Forest and Funnel Plot for PD group analysis. The overall effect size = 0.59 (0.21–0.97; p-value 0.00); Studies outside the funnel have publication bias, while studies inside and closer to the apex have higher methodological quality. PD: Probing Depth [7, 12, 30–59]

In Fig. 1, according to the Forest Plot to random-effects model, considering all groups, the overall effect size was 0.59 (0.21–0.97; p-value 0.00). The heterogeneity Tau-squared was 1.07, H-squared was 17.27 and I-squared was 0.94. In the test of overall effect size, z was equal to 3.08. The Funnel Plot also provides us with a rich analysis based on the standard error, that is, studies outside the funnel have publication bias, while studies inside and closer to the apex have higher methodological quality.

## CAL group

**Fig. 2:**
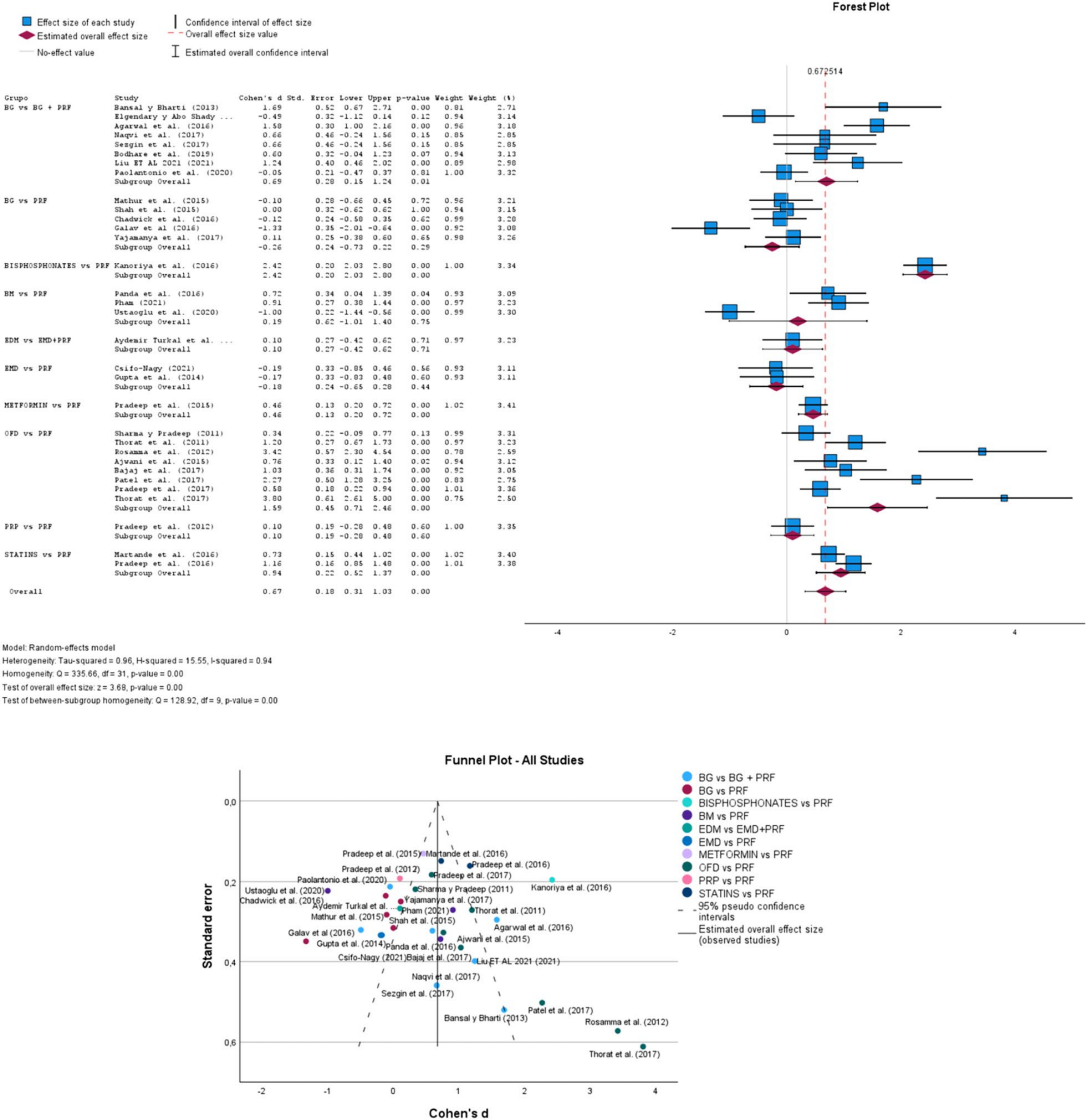
Forest and Funnel Plot for CAL group analysis. The overall effect size = 0.67 (0.31–1.03; p-value 0.00); Studies outside the funnel have publication bias, while studies inside and closer to the apex have higher methodological quality. CAL: Clinical Attachment Level [7, 12, 30–59]

In Fig. 2, according to the Forest Plot to random-effects model, considering all groups, the overall effect size was 0.67 (0.31–1.03; p-value 0.00). The heterogeneity Tau-squared was 0.96, H-squared was 15.55 and I-squared was 0.94. In the test of overall effect size, z was equal to 3.68. The Funnel Plot demonstrates evidence that the studies expressed publication bias. Pradeep et al. [56, 59] studies, and Martande et al. [51] found greater precision in their publication methodology based on 95% reliability.

## Bone group

**Fig. 3:**
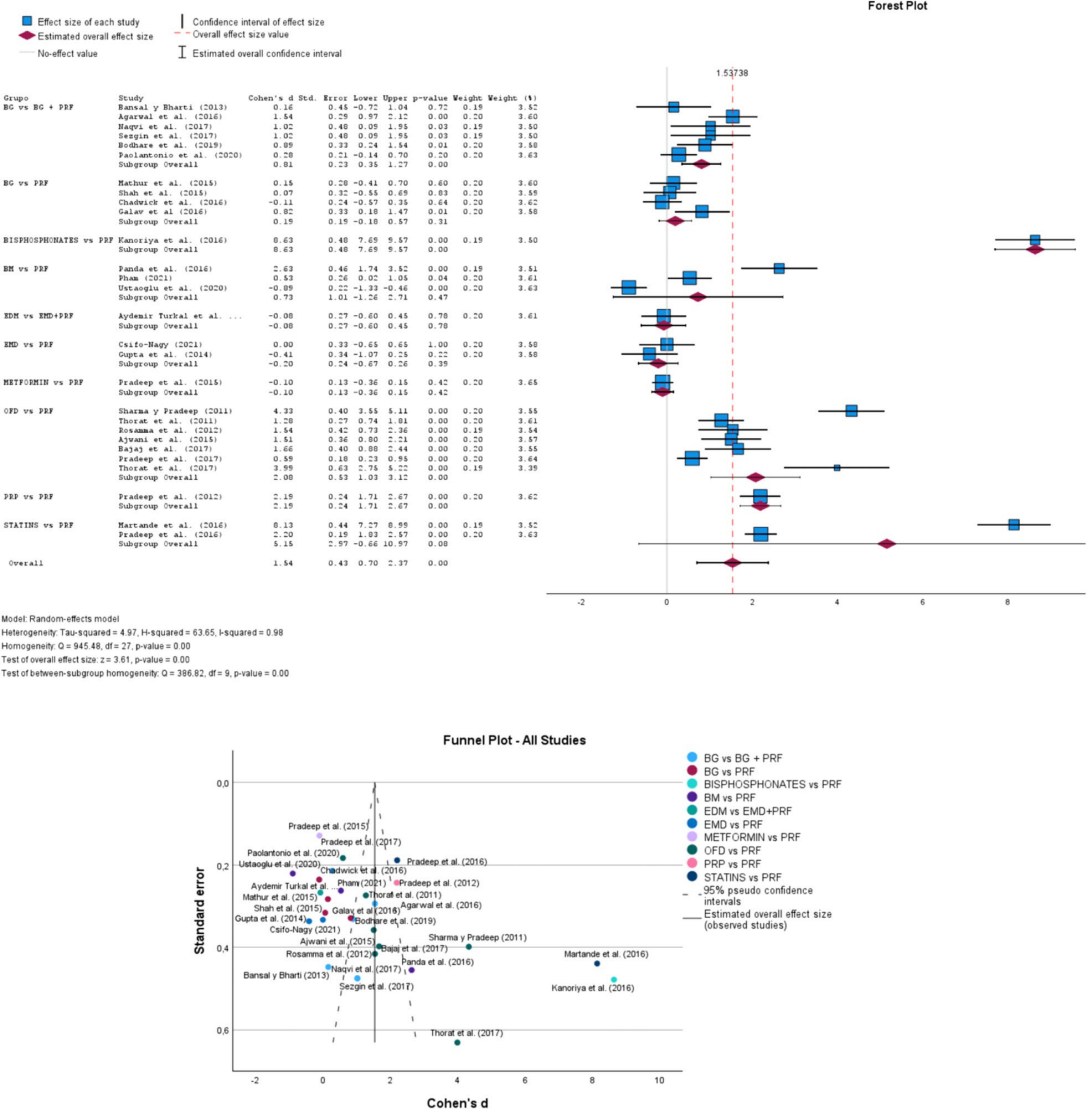
Forest and Funnel Plot for Bone group analysis. The overall effect size = 1.54 (0.70–2.37; p-value 0.00); Studies outside the funnel have publication bias, while studies inside and closer to the apex have higher methodological quality [7, 12, 30–59]

In Fig. 3, according to the Forest Plot to random-effects model, considering all groups, the overall effect size was 1.54 (0.70–2.37; p-value 0.00). The heterogeneity Tau-squared was 4.97, H-squared was 63.65 and I-squared was 0.98. In the test of overall effect size, z was equal to 3.61. The Funnel Plot, Thorat et al. [31], and Agarwal et al. [44] studies show greater precision in a CI95% when compared, for example, with Pradeep et al. [56].

## Network meta-analysis (NMA)

### PD group

**Fig. 4:**
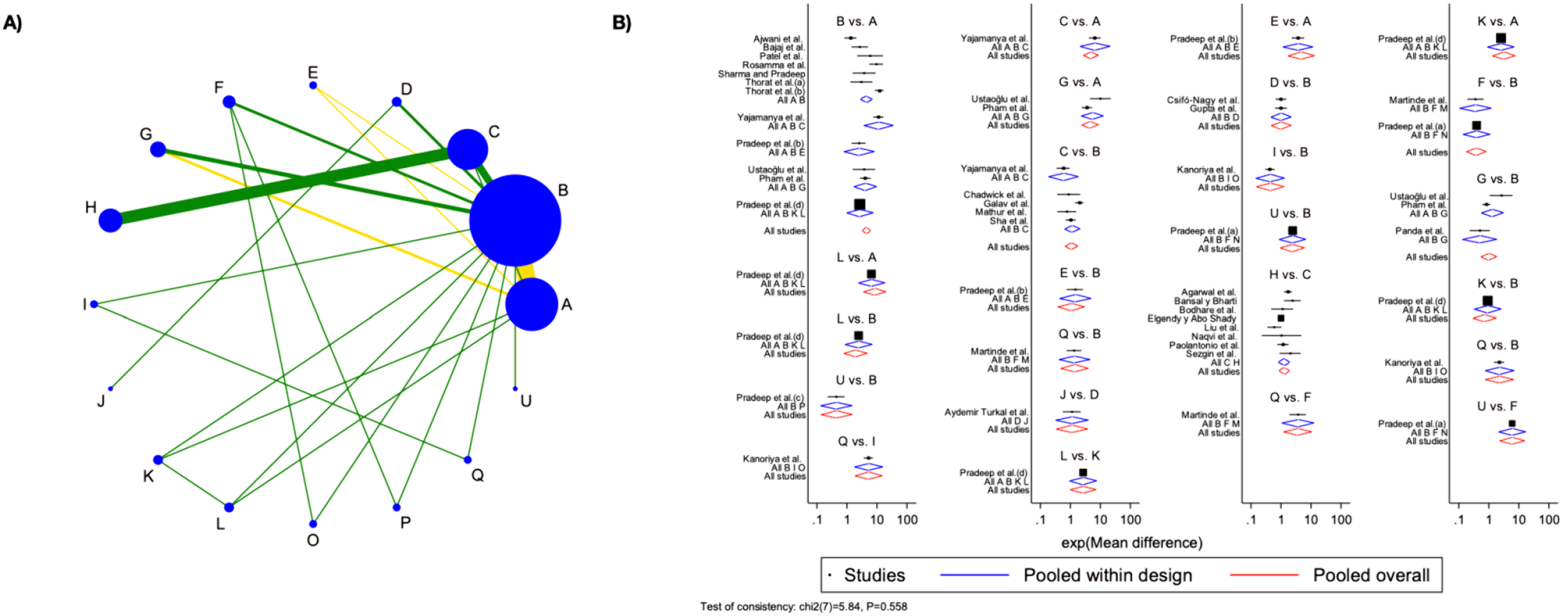
Network geometry plot for PD group analysis and Network forest plot for PD group analysis.** A** Most of the comparisons were at low risk of bias, however, the main one, PRF vs. blood clots presented moderate bias; **B** Overall inconsistency resulted in absence at global (p-value = 0.558) and local levels (p-value ranging between 0.139 to 0.997. The letters corresponds to: A = ROSU1.2% + PRF; B = BF1% + PRF; C = MF1% + PRF; D = Bone Fill + PRF; E = 1.2%ATV + PRF; F = EMD + PRF; G = Bone Fill; H = PRF + HA; I = EMD; J = BM; K = PRF; L = MF1%; O = PRP; P = BF; Q = ATV; U = Blood Clot. The acronym corresponds to: PRF: Platelet-Rich Fibrin; HA: Hydroxyapatite; BM: Barrier Membrane; EMD: Enamel Matrix Derivative; PRP: Platelet-Rich Plasma; BF: Bone Filler; MF: MetFormin; ROSU = Rosuvastin; ATV = Atorvastatin [7, 12, 30–59]

Thirty-two studies were considered for PD NMA and direct and indirect comparisons were represented in the network geometry plot. PRF was the most representative treatment group, followed by blood clots and bone filling (Fig. 4A).

Overall inconsistency resulted in absence at global (p-value = 0.558) and local level (p-value ranging between 0.139 to 0.997). Visualization of the inconsistency was figured in the network forest plot, in which effect sizes by study were graphically represented (Fig. 4B**)**.

Among the 15 interventions, 3 did not contribute to an improvement of PD, p-value > 0.05 (ATV, BF, and PRP), however, these interventions poorly contributed to the overall network geometry, with few studies being included. When also considering predictive intervals, of the 12 left interventions, 7 showed short predictive intervals, not crossing the null vertical line effect. These interventions might show positive results even in future studies and should be considered as an effective measure to improve PD compared to blood clots alone. When considering which intervention might be most effective, PRF was superior to ATV, BF, PRP, and MF1%, even though not statistically significant. EMD, BM, and bone fill reported very similar improvements in PD, with comparable effects to PRF. A combination of bone fill and PRF yielded better results compared to both bone fill and PRF alone, despite these results showing no statistical significance and large predictive intervals, which could lead in future clinical trials to null results. The combination of PRF with ATV, BF, MF, or rosuvastin (ROSU) generated increased effect size compared to PRF alone, while the addition of HA and EMD did not lead to gains (these comparisons were, however, not statistically significant).

Visualizing results based on SUCRA scores, the top 6 ranked treatments included a combination of PRF with other classes of drugs. PRF alone showed a SUCRA value of 49.3, representing almost 50% of cases with successful outcomes in terms of PD reduction. Similarly, bone filling registered a SUCRA value of 54.7, while a combination of PRF and bone fill raised the successful outcome at 68.7. Detailed SUCRA values and mean rank are illustrated in Supplementary Fig. 2.

## CAL group

**Fig. 5:**
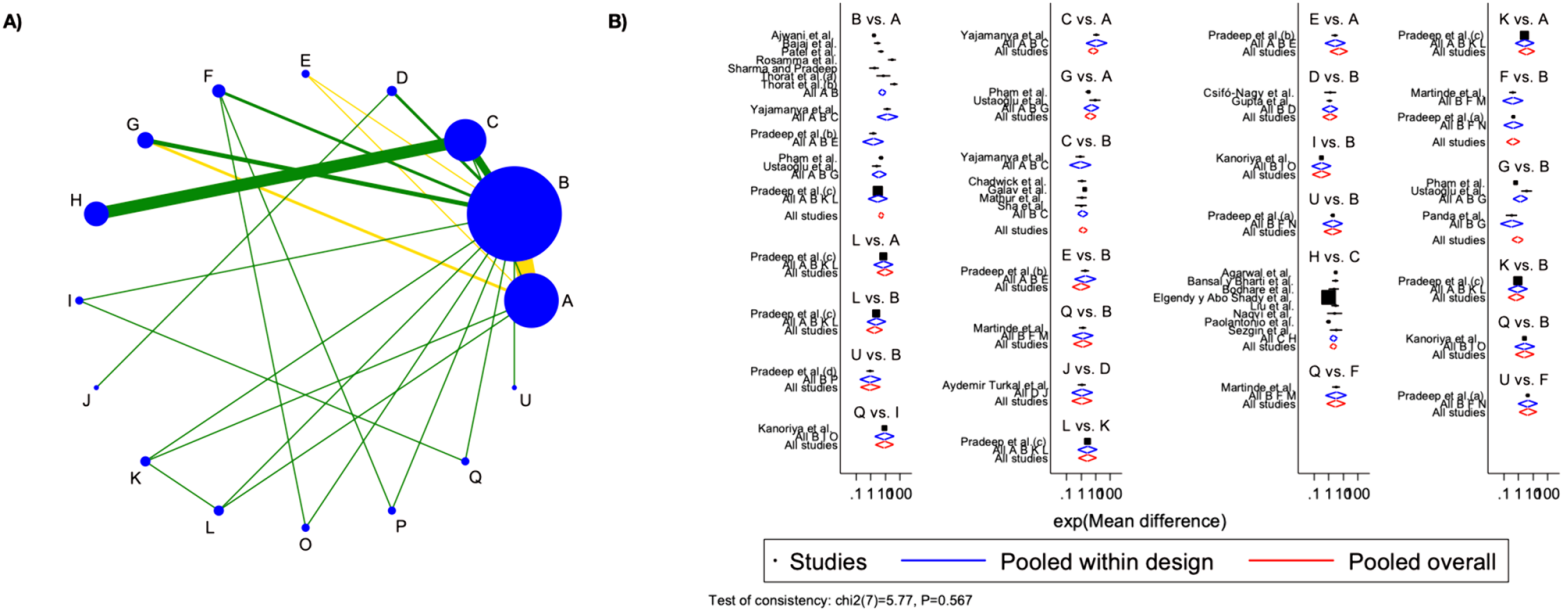
Network geometry plot for CAL group analysis and Network forest plot for CAL group analysis.** A** Most of the comparisons were at low risk of bias, however, the main one, PRF vs. blood clots presented moderate bias; **B** Overall inconsistency resulted in absence at global (p-value = 0.567) and local levels (p-value ranging between 0.102 to 0.996. The letters corresponds to: A = Bone Fill + PRF; B = BF1% + PRF; C = MF1% + PRF; D = 1.2%ROSU + PRF; E = EMD + PRF; F = 1.2%ATV + PRF; G = Bone Fill; H = EMD; I = PRF + HA; J = PRF; K = PRP; L = BM; O = MF1%; P = ATV; Q = BF; U = Blood Clot. The acronym corresponds to: PRF: Platelet-Rich Fibrin; HA: Hydroxyapatite; BM: Barrier Membrane; EMD: Enamel Matrix Derivative; PRP: Platelet-Rich Plasma; BF: Bone Filler; MF: MetFormin; ROSU = Rosuvastin; ATV = Atorvastatin [7, 12, 30–59]

Thirty-two studies were considered for CAL NMA and direct and indirect comparisons were represented in the network geometry plot (Fig. 5A). PRF was the most representative treatment group, followed by blood clots and bone filling.

Overall inconsistency resulted in absence at global (p-value = 0.567) and local level (p-value ranging between 0.102 to 0.996). Visualization of the inconsistency was figured in the network forest plot, in which effect sizes by study were graphically represented (Fig. 5B).

Among the 15 interventions, 5 did not contribute to an improvement of CAL, p-value > 0.05 (BF, ATV, MF1%, PRP, and EMD + PRF), however, these interventions poorly contributed to the overall network geometry, with few studies being included. When also considering predictive intervals, of the 10 left interventions, only a mixture of bone and PRF demonstrated short predictive intervals, not crossing the null vertical line effect. This intervention might show positive results even in future studies and should be considered as an effective measure to improve CAL compared to blood clots alone. When considering which intervention might be most effective, a combination of bone and PRF was considered against all the other treatments. This combination yielded superior statistically significant results compared to bone or PRF alone, as well as in comparison to ATV and BF. This combination kept higher CAL recovery even when compared to a mixture of PRF with ATV, EMD, MF, and ROSU, however not statistically significant. The addition of BF to PRF resulted in a similar CAL compared to the bone and PRF mixture.

Visualizing results based on SUCRA scores, top-ranked treatment resulted combination of bone and PRF, with a mean rank of three among all the interventions and a SUCRA value of 86.3. The fifth top-ranked treatment included a combination of PRF with other molecules, such as BF, MF, ROFU, EMD, and ATV. PRF alone showed a SUCRA value of 47.2, representing almost of 50% of cases with successful outcomes in terms of CAL reduction. Similarly, bone filling registered a SUCRA value of 59.8. Detailed SUCRA values and mean rank are illustrated in Supplementary Fig. 3.

## Bone group

**Fig. 6:**
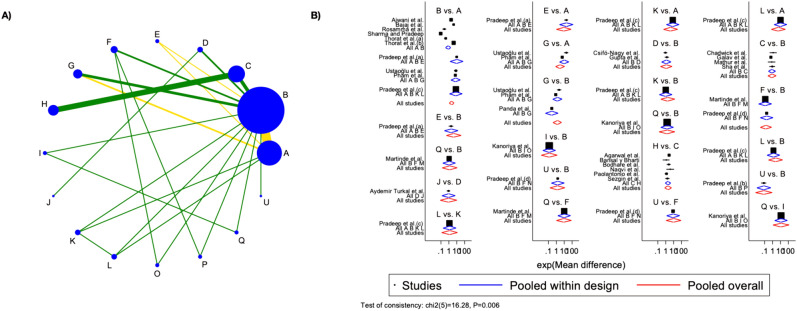
Network geometry plot for RBF group analysis and Network forest plot for RBF group analysis.** A** Most of the comparisons were at low risk of bias, however, the main one, PRF vs. blood clots presented moderate bias; (B) Overall inconsistency resulted in the present at the global level (p-value = 0.006), while at the local level, only blood clots versus BM and PRF versus BM yielded local inconsistency (p-values 0.012 and 0.033 respectively). The letters corresponds to: A = PRF + HA; B = MF1% + PRF; C = Bone Fill + PRF; D = MF1%; E = BF1%PRF; F = BM; G = 1.2%ATV + PRF; I = EMD; J = EMD + PRF; K = PRF; L = Bone Fill; O = Blood Clot; P = BF; Q = ATV; U = PRP. The acronym corresponds to: PRF: Platelet-Rich Fibrin; HA: Hydroxyapatite; BM: Barrier Membrane; EMD: Enamel Matrix Derivative; PRP: Platelet-Rich Plasma; BF: Bone Filler; MF: MetFormin; ROSU = Rosuvastin; ATV = Atorvastatin [7, 12, 30–59]

Twenty-eight studies were considered for RBF NMA and direct and indirect comparisons were represented in the network geometry plot (Fig. 6A). PRF was the most representative treatment group, followed by blood clots and bone filling.

Overall inconsistency resulted in present at the global level (p-value = 0.006), while at the local level, only blood clots versus BM and PRF versus BM yielded local inconsistency (p-values 0.012 and 0.033 respectively). Visualization of the inconsistency was figured in the network forest plot, in which effect sizes by study were graphically represented (Fig. 6B).

Among the 15 interventions, with surprise, 3 led to worse RBF (PRP, BF, and ATV). All the other treatments were characterized by improved RBF compared to blood clots, however, seeing the large predictive intervals, these interventions could lead to unsuccessful results in future trials. Only PRF and PRF combined with HA led to statistically significant RBF improvements since their confidence interval did not cross the line of null effect. PRF + HA led to the highest RBF and was statistically significant compared to PRP, BF, ATV, and blood clots. All the other interventions demonstrated lower RBF, however, compared to PRF + HA, these differences were not statistically significant.

Visualizing results based on SUCRA scores, top-ranked treatment resulted combination of PRF and HA, with a mean rank of 3.5 among all the interventions and a SUCRA value of 83.7. Of interest, MF and PRF + MF resulted in the best second and fourth interventions. In this case, a combination of bone with PRF led to top-ranked results, as the third-best intervention. Detailed SUCRA values and mean rank are illustrated in Supplementary Fig. 4.

## Discussion

### PD group

STATISTS vs. PRF has the largest sample size but is not significant between the study groups, however, we can specifically direct that the BIPHOSPHONATES vs. PRF, OFD vs. PRF, and PRF vs. PRF groups resulted in statistical significance with a p-value < 0.05. These results are evident at CI95% in the position of the diamond in the forest plot (Fig. 1) in each group of studies.

The group of studies BG vs. BG + PRF is not significant, but studies such as Bansal and Bharti [42], Agarwal et al. [44], and Kanoriya et al. [57] have statistically significant risk factors. However, Ustaoglu et al. [51] are significant as a protective factor. Martande et al. [58] and Pradeep et al. [59] are significant with a risk factor for the regenerative potential of PRF only in periodontal bone defects. Galav et al. [41] and Yajamanya et al. [12] are highly significant with protective risk for the use of ABG or PRF as a rebuilder of periodontal structures, and significant with a risk factor for radiographic results with PerioGlas and PRF. Pradeep et al. [56] is a significant study with a risk factor of the PRF group + MF1%. The studies of Sharma and Pradeep [30], Thorat et al. [31], Rosamma et al. [32], Bajaj et al. [34], Patel et al. [35], Pradeep et al. [59], and Thorat et al. [37] are highly significant studies with control risk. The Pradeep et al. [53] study is statistically significant for the control risk in the PD reduction and gain of CAL and BF (Fig. 1).

## CAL group

The results by groups of studies indicate that, according to Bilateral Significance, those less than 0.05 are statistically significant, for this the groups are BIPHOSPHONATES vs. PRF, STATISTAS vs. PRF, METFORMIN vs. PRF, OFD vs. PRF, as well as all the studies in a global way turn out to be significant using CAL.

In the analysis of results within the groups, we also found the studies that have statistical significance, in the BG vs. BG + PRF group the study Bansal and Bharti [42], and Agarwal et al. [44] where PRF and ADFDB are more effective than ADFDB with saline solution. Liu et al. where the BPBM-PRF complex is clinically more effective with effective results. BIPHOSPHANES vs. PRF Kanoriya et al. [57] was the focused therapy combining PRF + ALN 1% for the treatment of IBD in patients. In the BM vs. PRF group, the studies by Panda et al. [52] are significant with 72% sensitivity where PRF in combination with barrier membrane is more effective. Pham et al. [50] are significant with a p-value of 0.00 where PRF gave positive results for periodontal tissue. Ustaoglu et al. [51] are significant with a p-value of 0.00 where the PRF can give positive results as GTR in the treatment of IBD. In the group of STATISTS vs. PRF, we found that the study by Martande et al. [58] is significant with 73% of sensitivity to control risk (Fig. 2).

Pradeep et al. [59] are significant where OFD with ROSU 1.2% and PRF produce periodontal benefits. In the BONE vs. PRF group, the Galav et al. [41] study is statistically significant where ABG as PRF can be used predictably for the reconstruction of periodontal structures. In the METFORMIN vs. PRF group, Pradeep et al. [56] are significant with control risk where the PRF + MF 1% group was more effective than MF, PRF, or OFD. For the group of OFD vs. PRF studies, the studies of Thorat et al. [31], Rosamma et al. [32], Ajwani et al. [33], Bajaj et al. [34], Patel et al. [36], Pradeep et al. [59], Thorat et al. [37] are significant for control risk (Fig. 2).

## Bone group

The study groups were statistically significant due to the p-value of less than 0.05, in such a way that BG vs. BG + PRF, BIPHOSPHONATES vs. PRF, OFD vs. PRF, and PRP vs. PRF. At the global level of all the results of the study groups, significance is evidenced with a Z value of 3.605 and an effect size of 1.537.

When analyzing the Forest Plot graph (Fig. 3), it is evident that the overall result is relatively heterogeneous with an effect size of 1.537, with total risk. However when analyzing the groups of studies, although there are no significant results, the specific studies denote significance due to the results’ characteristics. In the BG vs. BG + PRF group, significance is evidenced in the study by Agarwal et al. [44], Naqvi et al. [45], Sezgin et al. [46], and Bodhare et al. [49].

For the BM vs. PRF group, the Panda et al. [52] and Pham [50] studies were significant with control risk, while the Ustaoglu et al. [51] study is significant with protective risk. In the STATISTA vs. PRF group, the studies of Martande et al. [58], and Pradeep et al. [59] were significant for control risk. In the BONE vs. PRF group, the study by Galav et al. [41] generated significance with a standard error of 0.33. In the group of OFD vs. PRF studies, the studies by Sharma and Pradeep [30], Thorat et al. [31], Rosamma et al. [32], Ajwani et al. [33], Bajaj et al. [34], Pradeep et al. [59], Thorat et al. [37] were significant as control risk. In the PRP vs. PRF group, the study by Pradeep et al. [46] is significant with control risk, with a standard error of 0.24 (Fig. 3).

## Conclusive NMA discussion

The present systematic review and meta-analysis suggest that using PRF to regenerate periodontal bone defects in humans may be effective compared to other regenerative techniques. However, it is important to note that results may vary depending on several factors, including the type of periodontal defect, the surgical technique used, and the quality of the studies included.

Despite its wide application in clinical practice, there is still controversy regarding which type of plasma concentrate can provide better results in relation to bone formation. Previous studies demonstrate that PC have some advantages, such as the formation of new trabecular bone, rapid resorption, and healing due to various growth factors. A combination of a material with low resorption, one that preserves the volume of the socket, together with another material that favors the formation of new bone, is supposed to be a good choice to promote osseointegration and primary stability. Other studies have also reported that alveolar ridge preservation (ARP) combined with any other material is superior to spontaneous healing [60].

The present study shows us that PRF use was the most representative treatment group in the treatment of PS, followed by blood clots and bone filling. However, the mixture of PRF with bone graft showed better results than the use of bone filler and PRF alone. However, these results do not present statistical significance and wide predictive intervals, which could result in null results in future clinical trials.

In the NBF group, it is possible to observe that the most effective intervention was the combination of bone and PRF. This combination produced statistically significant superior results compared to PRF or bone alone. The addition of BF to PRF resulted in a similar NBF with the combination of bone and PRF. However, the highest-rated treatment was the combination of bone and PRF, with an average rating of three across all interventions and a SUCRA value of 86.3.

## Limitations

One of the primary limitations of this systematic review and meta-analysis is the heterogeneity among the included studies. Variations in study design, patient demographics, types of periodontal defects, surgical techniques, and PRF preparation protocols may have contributed to significant heterogeneity in the results. This heterogeneity makes it challenging to draw definitive conclusions and may limit the generalizability of the findings. Despite efforts to include a wide range of relevant studies, publication bias remains a potential limitation. The quality of the included studies is crucial for the validity of the meta-analysis results. While efforts were made to assess the risk of bias in individual studies using the Cochrane Risk of Bias Tool, some studies may have inherent limitations that were not fully accounted for. The findings of this meta-analysis are based on a specific set of inclusion and exclusion criteria, including the restriction to studies conducted in humans, published in English, and involving non-smoking patients. This selectivity may limit the generalizability of the results to broader populations or clinical scenarios. The included studies may have used different PRF preparation protocols, including variations in centrifugation speed, duration, and the addition of activators. These differences in PRF preparation could impact the release of growth factors and other therapeutic components, potentially influencing treatment outcomes. Many of the included studies may have relatively short follow-up periods, which could limit our understanding of the long-term effectiveness and stability of PRF in periodontal regeneration. Ideally, longer-term follow-up data would provide a more comprehensive assessment of treatment success. The analysis primarily focused on probing depth (PD), clinical attachment level (CAL), and radiographic bone fill (RBF) as outcome measures. While these are essential parameters for assessing periodontal regeneration, other clinical and patient-reported outcomes may provide a more comprehensive evaluation of treatment success. Despite efforts to group studies based on similar interventions, there may still be clinical heterogeneity within the treatment groups. Variations in surgical techniques, operator skills, and patient compliance could introduce additional variability in the results.

## Conclusion

Based on the results of this systematic review and meta-analysis, the use of PRF appears to be a promising option for the regeneration of periodontal bone defects in humans. However, more high-quality randomized clinical trials are needed to confirm these findings and provide more precise guidance on the clinical use of PRF in dental practice.

## Supplementary Information

Below is the link to the electronic supplementary material.

Supplementary file1 (TIFF 12301 KB)

Supplementary file2 (TIFF 8101 KB)

Supplementary file3 (TIFF 8101 KB)

Supplementary file4 (TIFF 8101 KB)

Supplementary file5 (DOCX 16 KB)

Supplementary file6 (DOCX 24 KB)

Supplementary file7 (DOCX 22 KB)

## Data Availability

The data to support the findings of this study will be available on request from the corresponding author J.M.S.P.
