# Supplementary figures and images for: Regeneration of periodontal intrabony defects using platelet-rich fibrin (PRF): a systematic review and network meta-analysis
Source: Odontology. 2024 May 21;112(4):1047–68. doi: 10.1007/s10266-024-00949-7 (PMC11415441; doi:10.1007/s10266-024-00949-7)

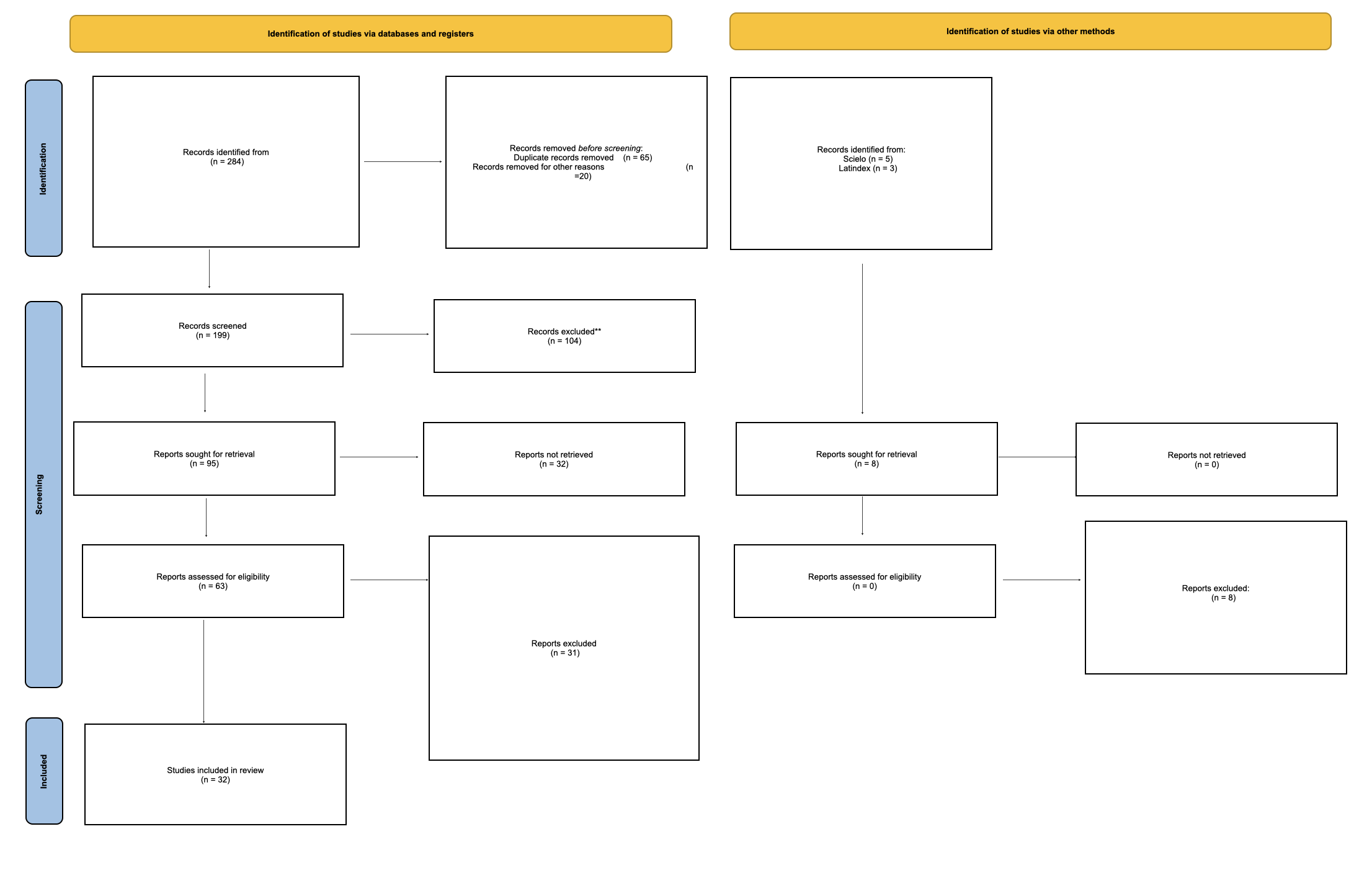

Supplement: Supplementary file 1 — Supplementary file1 (TIFF 12301 KB) [file 10266_2024_949_MOESM1_ESM.tiff]

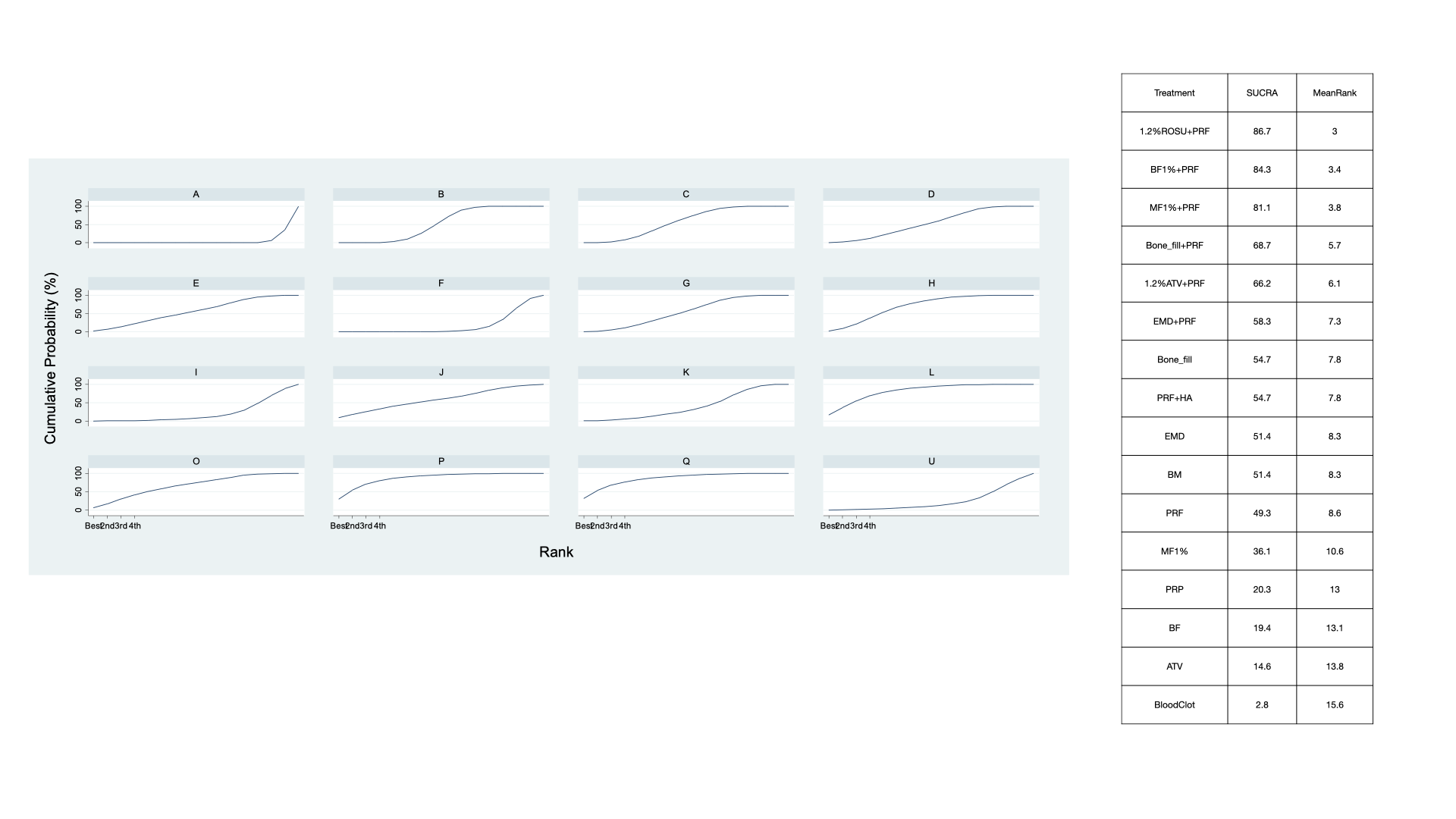

Supplement: Supplementary file 2 — Supplementary file2 (TIFF 8101 KB) [file 10266_2024_949_MOESM2_ESM.tiff]

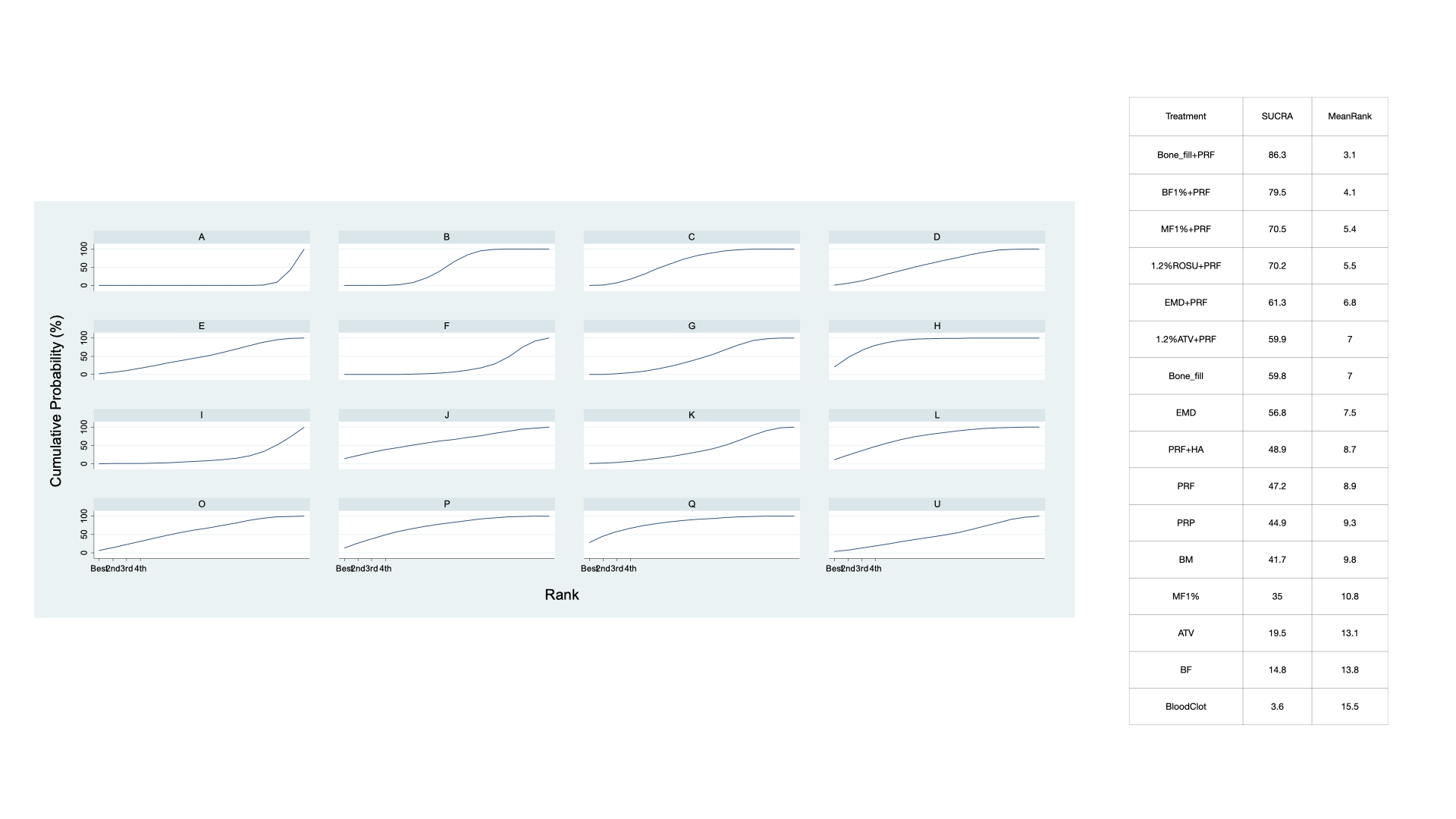

Supplement: Supplementary file 3 — Supplementary file3 (TIFF 8101 KB) [file 10266_2024_949_MOESM3_ESM.tiff]

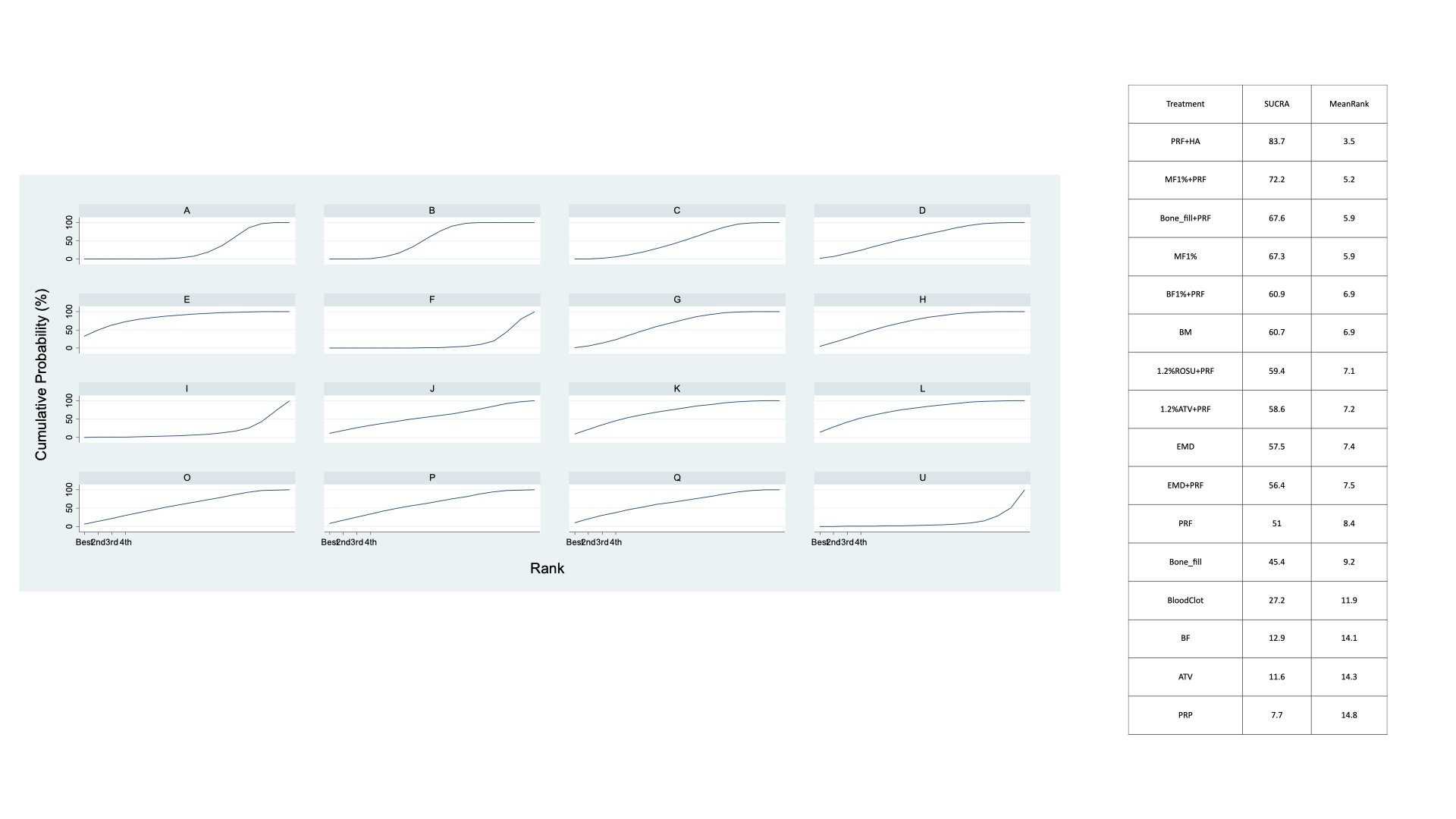

Supplement: Supplementary file 4 — Supplementary file4 (TIFF 8101 KB) [file 10266_2024_949_MOESM4_ESM.tiff]
