# Supplementary material for: Regeneration of periodontal intrabony defects using platelet-rich fibrin (PRF): a systematic review and network meta-analysis
Source: Odontology. 2024 May 21;112(4):1047–68. doi: 10.1007/s10266-024-00949-7 (PMC11415441; doi:10.1007/s10266-024-00949-7)
Supplement: Supplementary file 5 — Supplementary file5 (DOCX 16 KB) [file 10266_2024_949_MOESM5_ESM.docx]

**Supplementary Figures Captions**

**Supplementary Figure 1. Search strategy flow diagram.** Flow diagram following PRISMA 2020 guidelines for new systematic reviews which included searches of databases, registers, and other sources [21].

**Supplementary Figure 2. Detailed SUCRA for PD group analysis.** PRF alone showed a SUCRA value of 49.3. The letters corresponds to: A = 1.2%+PRF; B = BF1%+PRF; C = MF1%+PRF; D = Bone Fill+PRF; E = 1.2%ATV+PRF; F = EMD+PRF; G = Bone Fill; H = PRF+HA; I = EMD; J = BM; K = PRF; L = MF1%; O = PRP; P = BF; Q = ATV; U = Blood Clot. The acronym corresponds to: PRF: Platelet-Rich Fibrin; HA: Hydroxyapatite; BM: Barrier Membrane; EMD: Enamel Matrix Derivative; PRP: Platelet-Rich Plasma; BF: Bone Filler; MF: MetFormin; ROSU = Rosuvastin; ATV = Atorvastatin [7, 12, 30-59].

**Supplementary Figure 3. Detailed SUCRA for CAL group analysis.** PRF alone showed a SUCRA value of 47.2. The letters corresponds to: A = Bone Fill+PRF; B = BF1%+PRF; C = MF1%+PRF; D = 1.2%ROSU+PRF; E = EMD+PRF; F = 1.2%ATV+PRF; G = Bone Fill; H = EMD; I = PRF+HA; J = PRF; K = PRP; L = BM; O = MF1%; P = ATV; Q = BF; U = Blood Clot. The acronym corresponds to: PRF: Platelet-Rich Fibrin; HA: Hydroxyapatite; BM: Barrier Membrane; EMD: Enamel Matrix Derivative; PRP: Platelet-Rich Plasma; BF: Bone Filler; MF: MetFormin; ROSU = Rosuvastin; ATV = Atorvastatin [7, 12, 30-59].

**Supplementary Figure 4. Detailed SUCRA for RBF group analysis.** Top-ranked treatment resulted combination of PRF and HA, SUCRA value of 83.7. The letters corresponds to: A = PRF+HA; B = MF1%+PRF; C = Bone Fill+PRF; D = MF1%; E = BF1%PRF; F = BM; G = 1.2%ATV+PRF; I = EMD; J = EMD+PRF; K = PRF; L = Bone Fill; O = Blood Clot; P = BF; Q = ATV; U = PRP. The acronym corresponds to: PRF: Platelet-Rich Fibrin; HA: Hydroxyapatite; BM: Barrier Membrane; EMD: Enamel Matrix Derivative; PRP: Platelet-Rich Plasma; BF: Bone Filler; MF: MetFormin; ROSU = Rosuvastin; ATV = Atorvastatin [7, 12, 30-59].
