# Supplementary material for: Regeneration of periodontal intrabony defects using platelet-rich fibrin (PRF): a systematic review and network meta-analysis
Source: Odontology. 2024 May 21;112(4):1047–68. doi: 10.1007/s10266-024-00949-7 (PMC11415441; doi:10.1007/s10266-024-00949-7)
Supplement: Supplementary file 6 — Supplementary file6 (DOCX 24 KB) [file 10266_2024_949_MOESM6_ESM.docx]

**Table 1.** **General Information**

| **Author** | **Mean PD difference between baseline and final follow-up (mm)** | **Mean difference in CAL between baseline and final follow-up (mm)** | **Difference of means in bone between**  **start and end of follow-up (mm)** | **Centrifuge system** | **Volume of blood drawn (ml)** | **Centrifugation parameters**  **speed (rpm) × Time (min)** |
| --- | --- | --- | --- | --- | --- | --- |
| *OFD vs PRF* |  |  |  |  |  |  |
| Sharma and Pradeep 2011 [30] | 3.21 ± 1.64 (C) 4.55 ± 1.87 (T) | 2.77 ± 1.44 (C) 3.31 ± 1.76 (T) | 0.09 ± 0.11 (C) 2.50 ± 0.78 (T) | R-4C (REMI, Mumbai, India) | 10ml | 3000 × 10 |
| Thorat et al 2011 [31] | 3.56 ± 1.09 (C) 4.69 ± 1.45 (T) | 2.13 ± 1.71 (C) 4.13 ± 1.63 (T) | 1.24 ± 0.69 (C) 2.12 ± 0.69 (T) | - | - | 2700 × 12 |
| Rosamma et al. 2014 [32] | 2.40 ± 0.63 (C) 4.67 ± 0.90 (T) | 1.40 ± 1.06 (C) 4.73 ± 0.88 (T) | 0.64 ± 0.50 (C) 1.93 ± 1.07 (T) | KW-70 (Almicro Instruments, Haryana, India) | 10ml | 3000 × 10 |
| Ajwani et al. 2015 [33] | 1.60 ± 0.84 (C) 1.90 ± 0.74 (T) | 1.30 ± 0.68 (C) 1.80 ± 0.63 (T) | 0.80 ± 0.35 (C) 1.45 ± 0.50 (T) | R-4C (REMI, Mumbai, India) | 10ml | 3000 × 10 |
| Bajaj et al. 2017 [34] | 2.14 ± 1.26 (C) 3.14 ± 1.26 (T) | 1.59 ± 1.01 (C) 2.66 ± 1.07 (T) | 0.84 ± 0.99 (C) 2.24 ± 0.66 (T) | R-4C (REMI, Mumbai, India) | 10ml | 3000 × 10 |
| Patel et al. 2017 [35] | 2.40 ± 0.84 (C) 4.20 ± 1.69 (T) | 2.10 ± 0.74 (C) 3.70 ± 0.67 (T) | N/R | REMI-8C (REMI, Mumbai, India) | 10ml | 3000 × 10 |
| Pradeep et al. 2017 [36] | 2.97 ± 0.93 (C) 3.90 ± 1.09 (T1) 4.27 ± 0.98 (T2) | 2.67 ± 1.09 (C) 3.03 ± 1.16 (T1) 3.67 ± 1.03 (T2) | 0.93 ± 0.83 (C) 3.20 ± 0.89 (T1) 3.87 ± 1.33 (T2) | R-4C (REMI, Mumbai, India) | 10ml | 3000 × 10 |
| Thorat et al. 2017 [37] | 1.50 ± 0.34 (C) 4.00 ± 0.63 (T) | 0.33 ± 1.21 (C) 4.00 ± 0.63 (T) | 1.67 ± 0.06 (C) 3.09 ± 0.50 (T) | R-4C (REMI, Mumbai, India) | 10ml | 3000 × 12 |
| *BG vs PRF* |  |  |  |  |  |  |
| Mathur et al. (2015) [38] | 2.40 ± 1.06 (C) 2.67 ± 1.29 (T) | 2.67 ± 1.63 (C) 2.53 ± 1.06 (T) | 2.66 ± 1.84 (C) 2.93 ± 1.79 (T) | R-4C (REMI, Mumbai, India) | - | 3000 × 10 |
| Shah et al. 2015 [39] | 3.70 ± 0.68 (C) 3.67 ± 0.69 (T) | 2.97 ± 1.68 (C) 2.97 ± 1.56 (T) | 0.32 ± 1.59 (C) 0.42 ± 1.38 (T) | - | 10ml | 3000 × 10 |
| Chadwick et al. 2016 [40] | 2.00 ± 1.37 (C) 2.12 ± 1.41 (T) | 1.16 ± 1.33 (C) 1.03 ± 0.86 (T) | 1.53 ± 1.64 (C) 1.35 ± 1.60 (T) | Centrifuge 1310 (Salvin Dental Specialties, Charlotte, NC) | 10ml | 3000 × 10 |
| Galav et al. 2016 [41] | 4.80 ± 0.57 (C) 4.10 ± 0.63 (T) | 4.50 ± 0.52 (C) 3.90 ± 0.37 (T) | 4.10 ± 0.47 (C) 4.59 ± 0.70 (T) | - | 10ml | 3000 × 10 |
| Yajamanya et al. 2017 [12] | 3.68 ± 0.72 (C) 5.57 ± 1.10 (T1) 6.11 ± 0.92 (T2) | 4.14 ± 0.76 (C) 6.57 ± 1.45 (T1) 6.74 ± 1.55 (T2) | - | - | 10ml | 3000 × 10 |
| *BG vs BG+PRF* |  |  |  |  |  |  |
| Bansal and Bharti 2013 [42] | 3.10 ± 0.74 (C) 4.00 ± 0.82 (T) | 2.30 ± 0.70 (C) 3.40 ± 0.60 (T) | 1.93 ± 1.21 (C) 2.13 ± 1.28 (T) | - | 10ml | 3000 × 10 |
| Elgendy and Abo Shady 2015 [43] | 3.33 ± 0.36 (C) 3.30 ± 0.18 (T) | 3.55 ± 0.13 (C) 3.50 ± 0.06 (T) | - | - | 10ml | 3000 × 10 |
| Agarwal et al. 2016 [44] | 3.60 ± 0.51 (C) 4.15 ± 0.84 (T) | 2.61 ± 0.68 (C) 3.73 ± 0.74 (T) | 2.49 ± 0.64 (C) 3.50 ± 0.67 (T) | - | 10ml | 400×g ×12 |
| Naqvi et al. 2017 [45] | 3.15 ± 1.06 (C) 3.20 ± 2.30 (T) | 3.15 ± 1.06 (C) 4.10 ± 1.73 (T) | 5.70 ± 1.37 (C) 7.10 ± 1.37 (T) | - | 10ml | 3000 × 10 |
| Sezgin et al. 2017 [45] | 4.21 ± 1.21 (C) 4.93 ± 1.22 (T) | 3.27 ± 1.34 (C) 4.47 ± 1.60 (T) | 1.98 ± 0.80 (C) 2.55 ± 1.15 (T) | PC-02 (Process, Nice, France) | 10ml | 2700 × 12 |
| Liu et al. 2021 [47] | MOUTH PS 1.9 ± 0.7 (T)  2.4 ± 0.8 ( C)  LINGUAL PS 2.6 ± 0.8 (T)  3.2 ± 0.7 ( C) | MOUTH CAL 3.2 ± 0.6(T)   2.1 ± 1.1 ( C)  LINGUAL CAL  3.1 ± 0.8 (T)   2.1 ± 0.7 ( C) | 1.6% (0 to 5.9%) ( T)      2.1% (0 to 5.4%) ( C) | - | 10ml | 700 x 3 |
| Paolantonio et al. 2020 [47] | 4.214 ± 1.100 (T) 3.964 ± 1.035 ( C ) | 3.429 ± 0.741 (T) 3.286 ± 0.854 (C ) | 2.928 ± 0.716 (T) 2.678 ± 1.055 ( C) | - | 10ml | 3000 × 10 |
| Bodhare et al. 2019 [49] | 5.65 ± 1.66 (C) 5.75 ± 1.16 (T) | 4.20 ± 1.70 (C) 5.05 ± 1.09 (T) | 2.56 ± 0.95 (C) 3.51 ± 1.17 (T) | NR (REMI, Mumbai, India) | 10ml | 3000 × 10 |
| *BM vs PRF* |  |  |  |  |  |  |
| Pham 2021 [50] | 4.80± 0.71 (T) 4.63 ± 0.67 ( C1)  3.37 ± 1.00 ( C2) | 5.00 ± 0.46 (T) 4.53 ± 0.57 ( C1)  3.37 ± 1.22 Group 3 ( C2) | 45,25 ± 5,20 (T) 42,15 ± 6,39 ( C1) 13 ± 6,98 ( C2) | Hettich EBA 200, | 10ml | 2.500 X 14 |
| Ustaoğlu et al. 2020 [51] | 4.69±1.34(T) 5.67±1.21 (C 1), 3.36±1.12 (C2) | 4.19±1.05 (T) 5.50±1.53 (C1), 3.30±1.17 (C2) | 2.97±0.77(T) 3.85±1.16 ( C1) 0.9±0.80 (C2 ) | - | - | 2.800 x 12 |
| Panda et al. 2016 [52] | 3.19 ± 1.33 (C) 3.88 ± 1.15 (T) | 3.38 ± 1.45 (C) 4.44 ± 1.50 (T) | 0.80 ± 0.28 (C) 2.10 ± 0.64 (T) | Model C-854/6 (REMI, Mumbai, India) | 5ml | 3000 × 10 |
| *PRP vs PRF* |  |  |  |  |  |  |
| Pradeep et al. 2012 [53] | 2.97 ± 0.93 (C) 3.77 ± 1.07 (T1) 3.77 ± 1.19 (T2) | 2.83 ± 0.91 (C) 2.93 ± 1.08 (T1) 3.17 ± 1.29 (T2) | 0.13 ± 1.46 (C) 2.70 ± 0.79 (T1) 2.80 ± 0.89 (T2) | R-4C (REMI, Mumbai, India) | 10ml | 3000 × 10 |
| *EMD vs PRF* |  |  |  |  |  |  |
| Gupta et al. 2014 [54] | 1.80 ± 0.56 (C) 1.80 ± 0.77 (T) | 2.00 ± 0.54 (C) 1.87 ± 0.91 (T) | 2.08 ± 0.78 (C)  1.67 ± 1.17 (T) | NR (REMI, Mumbai, India) | 10ml | 3000 × 12 |
| CsifóNagy 2021 [7] | 4,67 ± 0,62 (T) 4,67 ± 0,62 C) | 2,60 ± 1,18 mm (C) 2,33 ± 1,58 mm (T) | 5.67 ± 0.89 mm (T) 5.67 ± 0.81mm C ) | Process for PRF Duo' (Choukroun) | 10ml | 1.300 x 8 |
| *EMD vs EMD + PRF* |  |  |  |  |  |  |
| Aydemir Turkal et al. 2016 [55] | 3.88 ± 1.26 (C) 4.00 ± 1.38 (T) | 3.29 ± 1.30 (C) 3.42 ± 1.28 (T) | 1.21 ± 1.24 (C) 1.13 ± 0.83 (T) | Mikro 22 R (Hettich, Tuttlingen, Germany) | 10ml | 400×g ×10 |
| *PRF vs PRF + metformin* |  |  |  |  |  |  |
| Pradeep et al. 2015 [56] | 3.00 ± 0.18 (C) 3.93 ± 0.25 (T1) 4.00 ± 0.18 (T2) 4.90 ± 0.30 (T3) | 2.96 ± 0.18 (C) 3.93 ± 0.25 (T1) 4.03 ± 0.18 (T2) 4.90 ± 0.30 (T3) | 0.49 ± 0.27 (C) 2.56 ± 0.28 (T1) 2.53 ± 0.30 (T2) 2.77 ± 0.30 (T3) | R-4C (REMI, Mumbai, India) | 10ml | 3000 × 10 |
| *PRF vs PRF + bisphosphonates* |  |  |  |  |  |  |
| Kanoriya et al. 2016 [57] | 2.86 ± 0.68 (C) 3.70 ± 0.91 (T1) 4.53 ± 0.81 (T2) | 3.03 ± 0.18 (C) 4.20 ± 0.66 (T1) 5.16 ± 0.46 (T2) | 0.38 ± 0.26 (C) 2.42 ± 0.21 (T1) 2.84 ± 0.26 (T2) | R-4C (REMI, Mumbai, India) | 10ml | 3000 × 10 |
| *PRF vs PRF + statins* |  |  |  |  |  |  |
| Martande et al. 2016 [58] | 2.76 ± 1.43 (C) 3.76 ± 1.12 (T1) 4.06 ± 1.22 (T2) | 2.50 ± 1.33 (C) 3.40 ± 1.13 (T1) 3.66 ± 1.42 (T2) | 0.27 ± 0.19 (C) 2.46 ± 0.33 (T1) 2.58 ± 0.36 (T2) | R-4C (REMI, Mumbai, India) | 5ml | 3000 × 12 to 14 |
| Pradeep et al. 2016 [59] | 3.10 ± 0.30 (C) 4.03 ± 0.18 (T1) 4.90 ± 0.31 (T2) | 2.47 ± 0.77 (C) 3.30 ± 0.65 (T1) 3.93 ± 0.78 (T2) | 1.43 ± 0.50 (C) 3.17 ± 0.65 (T1) 3.63 ± 0.67 (T2) | R-4C (REMI, Mumbai, India) | 10ml | 3000 × 10 |

Abbreviations: OFD: Open Flap Debridement; PRF: Platelet-Rich Fibrin; CAL: Clinical Attachment Level; T: Treatment; C: Control; EMD: Enamel Matrix Derivative; BM: Barrier Membrane; PRP: Platelet-Rich Plasma; PD: Probing Depth; BG: Bone Graft
