# Supplementary material for: Regeneration of periodontal intrabony defects using platelet-rich fibrin (PRF): a systematic review and network meta-analysis
Source: Odontology. 2024 May 21;112(4):1047–68. doi: 10.1007/s10266-024-00949-7 (PMC11415441; doi:10.1007/s10266-024-00949-7)
Supplement: Supplementary file 7 — Supplementary file7 (DOCX 22 KB) [file 10266_2024_949_MOESM7_ESM.docx]

| Study | **Selection bias** | | **Performance bias** | **Detection bias** | **Attrition bias** | **Reporting bias** | **Other biases** |
| --- | --- | --- | --- | --- | --- | --- | --- |
| **Author** | **Sequence Generation** | **Allocation concealment** | **Blinding of participants and personnel** | **Blinding of outcome assessors** | **Incomplete outcome data** | **Selective reporting of results** | **Other sources of bias** |
| Sharma and Pradeep 2011 [30] | Yes | Unclear | Yes | Yes | No | No | No |
| Thorat et al 2011 [31] | Yes | Unclear | Unclear | Yes | No | No | No |
| Rosamma et al.   2014 [32] | Yes | Unclear | Unclear | Yes | No | No | No |
| Ajwani et al. 2015 [33] | Yes | Unclear | Unclear | Yes | No | No | No |
| Bajaj et al. 2017 [34] | Yes | Unclear | Yes | Yes | No | No | No |
| Patel et al. 2017 [35] | Yes | Unclear | Yes | Yes | No | No | No |
| Pradeep et al. 2017 [36] | Yes | Unclear | Yes | Yes | No | No | No |
| Thorat et al.  2017 [37] | Yes | Unclear | Unclear | Yes | No | No | No |
| Mathur et al. 2015 [38] | Yes | Yes | Yes | Yes | No | No | No |
| Sha et al.  2015 [39] | Yes | Yes | Yes | Yes | No | No | No |
| Chadwick et al. 2016 [40] | Yes | Yes | Yes | Yes | No | No | No |
| Galav et al.  2016 [41] | Yes | Yes | Yes | Yes | No | Yes | No |
| Yajamanya et al.  2017 [12] | Yes | Yes | Yes | Yes | No | No | No |
| Bansal and Bharti 2013 [42] | Yes | Yes | Yes | Yes | No | No | No |
| Elgendy and Abo Shady 2015 [43] | Yes | Yes | Yes | Yes | Unclear | No | No |
| Agarwal et al.  2016 [44] | Yes | Unclear | Yes | Yes | No | No | No |
| Naqvi et al.   2017 [45] | Yes | Yes | Yes | Yes | No | No | No |
| Sezgin et al. 2017 [46] | Yes | Yes | Yes | Yes | No | No | No |
| Liu et al. 2021 [47] | Yes | Unclear | Yes | Yes | Unclear | No | No |
| Paolantonio et al. 2020 [48] | Yes | Unclear | Yes | Yes | No | No | No |
| Bodhare et al.  2019 [49] | Yes | Yes | Yes | Yes | No | No | No |
| Pham 2021 [50] | Yes | Yes | Yes | Yes | No | No | No |
| Ustaoğlu et al.   2020 [51] | Yes | Unclear | Unclear | Yes | No | No | No |
| Panda et al.   2016 [52] | Yes | Yes | Yes | Yes | No | No | No |
| Pradeep et al. 2012 [53] | Yes | Unclear | Yes | Yes | No | No | No |
| Gupta et al. 2014 [54] | Yes | Yes | Yes | Yes | No | No | No |
| CsifóNagy  2021 [7] | Yes | Yes | Yes | Yes | No | No | No |
| Aydemir Turkal et al. 55   2016 | Yes | Yes | Yes | Yes | No | No | No |
| Pradeep et al. 2015 [56] | Yes | Unclear | Yes | Yes | No | No | No |
| Kanoriya et al.  2016 [7] | Yes | Yes | Yes | Yes | No | No | No |
| Martinde et al. 2016 [58] | Yes | Yes | Yes | Yes | No | No | No |
| Pradeep et al. 2016 [59] | Yes | Unclear | Yes | Yes | No | No | No |
| Positive (good) indicator | | Unclear | | Negative (bad) indicator | |  |  |
